# Trends in the incidence of primary liver cancer in Central Uganda, 1960–1980 and 1991–2005

**DOI:** 10.1038/sj.bjc.6604893

**Published:** 2009-01-27

**Authors:** P Ocama, S Nambooze, C K Opio, M S Shiels, H R Wabinga, G D Kirk

**Affiliations:** 1 Department of Medicine, Makerere University, PO Box 7072, Kampala, Uganda; 2 Department of Pathology, Makerere University, PO Box 7072, Kampala, Uganda; 3 Department of Epidemiology, Johns Hopkins Bloomberg School of Public Health, 615 N Wolfe St, Baltimore, MD 21205, USA

**Keywords:** primary liver cancer, hepatocellular carcinoma, cancer registry, Africa, gender differences, human immunodeficiency virus/acquired immunodeficiency syndrome (HIV/AIDS)

## Abstract

Primary liver cancer (PLC) incidence trends from Africa are unknown. Using Kampala Cancer Registry data from 1960 to 1980 and 1991 to 2005, we identified 771 PLCs. Although rates were stable among men, PLC incidence among women increased >50%. Investigations of viral hepatitis, aflatoxin, obesity, and human immunodeficiency virus (HIV) may help to explain the increasing incidence of hepatocellular carcinomas (HCCs).

Trends in primary liver cancer (PLC) incidence rates will generally reflect temporal changes in exposure to aetiological agents. Worldwide, and in sub-Saharan Africa in particular, the great majority of PLCs are hepatocellular carcinomas (HCCs). In North America and Europe at present, HCCs are one of the few cancers observed with increasing incidence (Taylor-Robinson *et al*, 1997; El-Serag, 2004; West *et al*, 2006), largely attributed to earlier exposure to hepatitis C virus (HCV) (Davila *et al*, 2004). Prevalence of obesity and diabetes has also been increasing in these populations during concurrent time periods and has been suggested as another possible aetiological factor in rising HCC rates (Calle *et al*, 2003; El-Serag *et al*, 2004).

However, trends in HCC rates from other regions are less clear. In particular, limited data exist from regions where HCCs is primarily attributable to chronic hepatitis B viral infection. In Asia, HCC rates may be declining (Goh, 1997; McGlynn *et al*, 2001). Reductions in HCC incidence among young children in Taiwan has been linked to nation-wide hepatitis B vaccination (Chang *et al*, 1997). In sub-Saharan Africa, hepatitis B viral infection is endemic and the attributable fraction of HCCs due to hepatitis B virus (HBV) is high (∼60%) (Kirk *et al*, 2004; Parkin, 2006). Further, most African countries do not routinely provide or only recently initiated hepatitis B vaccination as part of their national immunisation programmes. Urbanisation, obesity, and HIV infection might also affect HCC rates in Africa. To characterise temporal trends in HCC rates within an urban African population, we evaluated cancer registry data collected in Central Uganda from 1960 through 2005.

## Materials and methods

### Kampala Cancer Registry

The Kampala Cancer Registry (KCR) was established in the Department of Pathology, College of Health Sciences, Makerere University as a population-based cancer registry for Kyadondo County, Uganda, in 1951. Data were not complete during the earliest years and registration was halted in the 1980's because of political instability. The Kampala Cancer Registry cancer registration methods have been described earlier (Wabinga *et al*, 2000). Briefly, information on demographics, type of cancer (coded using the second edition of the International Classification of Diseases for Oncology (Percy *et al*, 1990)), diagnostic method, and incidence date for all cancer cases was collected from the major health units and histopathology laboratories throughout Kyadondo County through active and passive methods. Data processing is done by CANREG, a computerised cancer registration system (Cooke, 1998) in use since 1989, with entry of earlier manually collected data. Data from 1960 to 1980 and 1991 to 2005 were considered complete for the present analyses. Our analysis of de-identified registry data was not considered human-subjects research and exempted from IRB review.

### Case definitions

On the basis of histology, PLC cases were categorised into HCCs, non-HCCs, and malignancy not otherwise specified (NOS). Malignancies NOS (*n*=267) accounted for 34% of the cases; nearly all (94%) were diagnosed clinically without histological confirmation. Concurrent with increased reliance on ultrasonography and *α*-fetoprotein for HCCs clinical confirmation, the proportion of PLCs histologically confirmed as HCCs declined recently compared with the earlier time period (86–69% in men; 90–67% in women). To estimate HCC trends appropriately, we assumed that many malignancies NOS would have been classified as HCCs, had histological confirmation been carried out. Therefore, we calculated the proportion of PLC that were histologically confirmed HCCs by sex and time period and applied these proportions to the malignancies NOS; for HCC incidence estimates, these cases were assumed to be HCCs.

### Statistical analysis

The age-standardised incidence rates (ASRs) for PLC and for HCCs, stratified by sex, were estimated in two broad time periods (1960–1980 and 1991–2005) and six shorter intervals (1960–1966, 1967–1971, 1972–1980, 1991–1994, 1995–1997, and 1998–2005). Calendar periods were defined on the basis of a grouping used in an earlier publication (Wabinga *et al*, 2000). Substantial population growth occurred in Kyadondo County from 268 500 residents in 1960 to 1 911 720 in 2005; the proportion under 45 years of age increased slightly from 89.2 to 94.0% during this period. To calculate incidence rates, annual sex- and age-specific (within 5-year groups) population denominator estimates were interpolated based on the 1959, 1969, 1980, 1991, and 2005 census estimates, assuming constant growth rates between enumerations (Wabinga *et al*, 2000). Incidence rates were standardised to age- and sex-specific world population estimates for 2000 (US Census Bureau, 2007). Owing to missing age at diagnosis, 27 cases were excluded from incidence rate calculations. ASRs and standard errors were calculated (Boyle and Parkin, 1991) with estimation of 95% confidence intervals (CIs). Incidence rate ratios (IRRs) with 95% CIs were calculated for comparison of rates by calendar period and gender. Analyses were performed using Microsoft Excel 2003 and Intercooled Stata 8.2 (College Station, TX, USA).

## Results

The Kampala Cancer Registry recorded 771 PLCs during the study, including 261 (33.9%) from 1960 to 1980 and 510 (66.1%) from 1991 to 2005. Among the PLC cases, the median age at diagnosis was 45 years (interquartile range (IQR)=33, 60; range 1–97) and the male-to-female ratio was 2.1. Hepatocellular carcinoma was by far the predominant tumour type, comprising 79% of all histologically confirmed PLC. Demographic characteristics for PLC and for HCC were compared across time periods (Table 1). From 1960–1980 to 1991–2005, the median age at histologically confirmed HCC diagnosis increased slightly from 42 to 44 years. The proportion of histologically confirmed HCCs diagnosed among women doubled in the latter period compared with the earlier time period with the ratio of males to females decreasing (3.9–1.4).

Incidence rates of PLC and HCC among men were relatively stable from 1960–1980 to 1991–2005 (Table 2). However, among women, HCC incidence increased >50% during this period (IRR=1.56; 95% CI 1.13–2.16). The IRRs comparing men to women decreased from 2.32 (95% CI 1.70–3.17) in 1960–1980 to 1.30 (95% CI 1.04–1.63) in 1991–2005. Findings for PLC were similar to those for HCC (Table 2).

**Figure 1:**
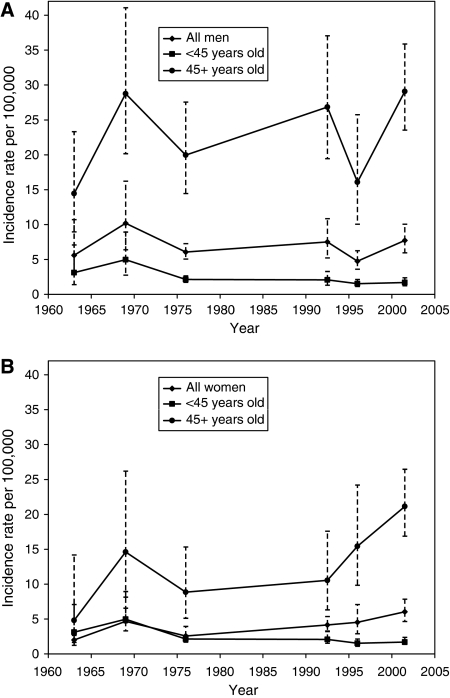
Age-standardised incidence rates of primary liver cancer among men (**A**) and women (**B**) overall, and stratified by age 45 years and older in Kyadondo County, Uganda: 1960–2005. Bars represent 95% confidence intervals, calculated using a natural logarithmic transformation, around each estimate.

When PLC incidence was examined within smaller calendar periods, trends of increasing HCC rates were observed among both the sexes, but the increase was substantially more apparent among women (Figure 1). For both sexes, the greatest increase in PLC incidence was observed in older age groups (Figure 1), increasing around twofold among men and fourfold among women aged 45 years or older.

## Discussion

In an analysis of cancer registry data compiled over five decades, we describe PLC and HCC incidence rates in urban Kampala, Uganda, which are stable among men but increasing among women. The Kampala Cancer Registry provides population-based HCC incidence estimates over a much longer time period than reported earlier from Africa (Bah *et al*, 2001). Uniquely, these data allow examination of temporal trends and prompt exploration for underlying changes in HCC etiological agents to explain observed changes in incidence.

Although well-controlled studies of HCC etiology in Uganda have not been carried out, a large proportion will be attributable to chronic hepatitis B viral infection (Tabor *et al*, 1977; Parkin, 2006). In contrast to some Asian HBV-endemic countries where HCC incidence rates may be declining (Goh, 1997; McGlynn *et al*, 2001), our data suggest that HCC rates are increasing in Uganda. National estimates of chronic HBV prevalence in Ugandan adults is 9% (MOH-Uganda, 2006). Comparing studies from earlier decades (Sadikali, 1972; Tabor *et al*, 1977; de Lalla *et al*, 1990) to the more recent ones (Nakwagala and Kagimu, 2002; Pido and Kagimu, 2005), no compelling evidence exists that HBV prevalence has changed substantively in Uganda. Notably, the male-to-female ratio in HBV-related HCCs would be expected to increase rather than decrease as we observed. Importantly, Uganda incorporated childhood vaccination against hepatitis B into their national immunisation programme in 2002, so any impact of this highly effective preventive intervention on HCC rates will not likely be observable for years.

Although increasing HCC rates in Europe and North America are largely attributed to HCV, chronic HCV infection is very uncommon in Uganda (Hladik *et al*, 2006). Chronic HCV infection with viremia is rare even in high-risk persons (Jackson *et al*, 1991; Biggar *et al*, 2006). Although it is unlikely that HCV would fully explain the increasing HCC rates, further evaluation is warranted, particularly because PLC increases among older Ugandans are consistent with an HCV cohort effect reported from other sub-Saharan African populations (Kirk *et al*, 2006).

Dietary exposure to aflatoxin is a well-recognised cause of HCC, including an early report from Uganda (Alpert *et al*, 1971). Although a variety of Ugandan foods may be contaminated with aflatoxin (Kaaya and Kyamuhangire, 2006), human exposures have not been assessed. Rural to urban migrants in South Africa retained higher HCC risk, attributed in part to greater aflatoxin exposure (Kew *et al*, 1983; Kimbi *et al*, 2005). During recent decades, Kampala has been dramatically shaped by a large influx of persons migrating from rural areas.

Recent data have linked obesity and diabetes to HCC development (Calle *et al*, 2003; El-Serag *et al*, 2004; Larsson and Wolk, 2007). In Uganda, reliable population data on changes in obesity are sparse. However, in light of the relative increase in HCC among women, it is interesting to note that Ugandan women were more likely to show the association between overweight and type 2 diabetes than the generally leaner men (Lasky *et al*, 2002).

Data are limited on the prevalence or trends of other factors potentially influencing HCC rates. Per capita alcohol consumption in Uganda is among the highest in the world (WHO, 2004). Anecdotal evidence suggests that drinking alcohol and cigarette smoking by women have become more acceptable and increased in Uganda during recent years. Reduced parity is associated with increasing HCC risk among women (Yu *et al*, 2003). According to the United Nations Population Division data, total fertility rates in Uganda appear to have been stable from 1970 to 1990, with subsequent small annual declines of around 0.5% from 1990 to 2006.

The major demographic and health impact occurring in Uganda between 1960 and 2005 has clearly been the human immunodeficiency virus/acquired immunodeficiency syndrome (HIV/AIDS) epidemic. Although HIV will accelerate HBV or HCV disease progression, HIV-infected persons rarely survive long enough to manifest HCC in the absence of highly active antiretroviral therapy (HAART). Owing to the competing risks of AIDS, studies in the United States failed to show HCC increases among HIV-infected persons through the early HAART years (Mbulaiteye *et al*, 2003; Kramer *et al*, 2005). However, recent studies with longer follow-up have documented significant increases in liver disease (Bica *et al*, 2001; Salmon-Ceron *et al*, 2005) and suggested HCC increases (Clifford *et al*, 2005; Powles *et al*, 2006). Similar to pre-HAART data from developed countries, earlier Ugandan studies have not shown an increased HCC risk associated with HIV (Wabinga *et al*, 2000; Mbulaiteye *et al*, 2006). HAART was introduced in Uganda in 2003–2004; therefore, neither those earlier studies nor the current analysis would reflect HAART effects. Our evidence that HCC rates appear to be increasing before any observable HAART-related effect raises considerable concern that the burden of HCCs may expand substantially in future years (Powles *et al*, 2006).

Limitations of cancer registry data collected in a resource-limited settings have been discussed in detail earlier (Wabinga *et al*, 2000). In brief, the completeness of KCR cancer registration was formally estimated at 93% for HCC cases (Parkin *et al*, 2001). However, observed HCC rates were lower than in some other HBV-endemic regions, suggesting possible under-ascertainment. We did observe temporal changes in HCC diagnostic methods with decreased reliance on liver biopsy. However, in HBV-endemic regions the HCC case definition utilising clinical criteria, ultrasound, and α-fetoprotein has high predictive accuracy and excellent specificity (Ho and Johnson, 1999). Although concerns regarding the accuracy of population denominator estimates interpolated from periodic census enumerations can be raised, it seems unlikely that estimates would have been differential by gender to account for the observed sex differences.

In conclusion, we present long-term cancer registry data from Uganda suggesting stable HCC incidence among men with increasing rates among women. With the potential for increasing HCC rates after prolonged survival of effectively treated HIV-infected persons, continued HCC surveillance will be vital to monitor trends. Irrespective of gender, HCCs present at very advanced stages and are highly lethal (Gondos *et al*, 2005). Therefore, systematic investigation of HCC etiological factors in Uganda is needed to inform appropriate primary preventive interventions (e.g., hepatitis B vaccine, aflatoxin reduction programmes, dietary, or physical activity interventions to prevent obesity).

## Figures and Tables

**Table 1:** Demographic characteristics of primary liver cancers and histologically confirmed hepatocellular carcinoma cases in the Kampala Cancer Registry, Kyadondo County, Uganda: 1960–1980 and 1991–2005

	**Primary liver cancer cases**		**Hepatocellular carcinoma cases**	
Time period	1960–1980	1991–2005	1960–1980	1991–2005
*N* (%)	261	510	201	120
Median age (IQR)^a^	42 (32, 55)	48 (33, 61)	42 (34, 53)	44 (30, 54)
				
*Sex* (%)				
Male	205 (79%)	296 (58%)	160 (80%)	69 (58%)
Female	56 (21%)	214 (42%)	41 (20%)	51 (43%)
Male : female ratio	3.7	1.4	3.9	1.4

a IQR=interquartile range.

**Table 2:** Age-standardised incidence rates for primary liver cancer (PLC) and hepatocellular carcinoma (HCC) by gender in Kyadondo County, Uganda: 1960–1980 and 1991–2005

	**1960–1980**			**1991–2005**				
	**N**	**ASRs^a^**	**95% CIs**	**N**	**ASRs**	**95% CIs**	**IRR:recent to early period**	**95% CIs**
*PLC*								
Males	195	6.96	5.88–8.04	288	7.13	6.17–8.09	1.02	0.83–1.26
Females	53	2.93	2.09–3.77	208	5.33	4.51–6.15	1.82	1.36–2.44
IRR:males to females		2.38	1.77–3.19		1.34	1.09–1.64		
								
*HCC*								
Males	173	6.15	5.13–7.17	218	5.38	4.55–6.20	0.87	0.71–1.08
Females	49	2.65	1.85–3.44	156	4.14	3.42–4.86	1.56	1.13–2.16
IRR:males to females		2.32	1.70–3.17		1.30	1.04–1.63		

a ASRs=age-standardised incidence rates, calculated per 100 000 person-years; CIs=confidence intervals; IRR=incidence rate ratio.
